# A double-blind, randomized, crossover trial protocol of whole hemp seed protein and hemp seed protein hydrolysate consumption for hypertension

**DOI:** 10.1186/s13063-020-4164-z

**Published:** 2020-04-23

**Authors:** Maryam Samsamikor, Dylan Mackay, Rebecca C. Mollard, Rotimi E. Aluko

**Affiliations:** 1 Department of Food and Human Nutritional Sciences, University of Manitoba, Winnipeg, MB R3T 2N2 Canada; 2 The Richardson Centre for Functional Foods and Nutraceuticals, University of Manitoba, 196 Innovation Drive, Winnipeg, MB R3T 2N2 Canada

**Keywords:** Casein, Hemp seed protein, Bioactive peptides, Hypertension

## Abstract

**Background:**

Primary hypertension accounts for almost 95% of all cases of high blood pressure and is a major modifiable risk factor for cardiovascular diseases. Lifestyle interventions have been shown to prevent hypertension. One of the prominent potential therapeutic lifestyle strategies to prevent or manage hypertension is increasing dietary protein as a macronutrient or as bioactive peptides. An emerging plant-based protein source that may have anti-hypertensive properties is hemp seed.

**Methods/design:**

A randomized, double-blind, crossover clinical trial will be conducted on 35 hypertensive participants aged 18–75 years, with a BMI between 18.5 and 40 kg/m^2^, systolic blood pressure (SBP) between 130 and 160 mmHg and diastolic blood pressure (DBP) ≤ 110 mmHg. The trial will be conducted for a period of 22 weeks and will consist of three treatment periods of 6 weeks, separated by 2-week washout periods. The treatments will be consumed twice a day and consist of 25 g casein, hemp seed protein (HSP), or HSP plus HSP hydrolysate (HSP+). The primary outcome of this trial is 24-h SBP, measured on the first day of first phase and the last day of each phase. Office-measured blood pressure, pulse-wave velocity and augmentation index and anthropometrics will be determined at the first and last days of each period. Also, body composition will be assessed by dual x-ray absorptiometry (DXA) scan on the first day of the first phase and within the last 2 days of each treatment period. Blood samples will be collected on the first and last 2 days of each treatment phase whereas urine samples will be collected on the first day of the first phase plus the last day of each phase to be analyzed for specific biomarkers.

**Discussion:**

This trial protocol is designed to evaluate the hypotensive potential of consuming whole HSP, and HSP+, in comparison to casein protein. This study will be the first trial investigating the potential anti-hypertensive benefit of dietary hemp protein plus bioactive peptide consumption in humans.

**Trial registration:**

National Clinical Trial (NCT), ID: NCT03508895. Registered on 28 June 2018. Retrospectively registered on the publicly accessible Registry Databank at ClinicalTrials.gov (http://ClinicalTrials.gov).

## Background

Hypertension afflicts over 7.3 million Canadians [1] and is the number-one modifiable risk factor for death and disability globally [2], accounting for 13.5% of all global premature deaths [3]. Over the last 25 years, treatments to control hypertension have increased markedly in Canada [4]; however, one third of the diagnosed hypertensive population remains uncontrolled and many people remain undiagnosed. Uncontrolled hypertension can lead to a spectrum of diseases including stroke, coronary artery disease, as well as heart and kidney failure [5].

The exact etiology of the development and progression of primary hypertension remains uncertain; however, both genetic and lifestyle factors, including diet in particular, have been implicated. Many interconnected contributing factors have been proposed to explain the increase in blood pressure (BP) leading to hypertension. Factors include over-activation of the renin-angiotensin-aldosterone system (RAAS), endothelial dysfunction, elevated sympathetic activation, and other genetic predispositions [6–8]. Current therapeutic agents for BP control have proven to be reasonably effective; however, the incidence of mortality due to high BP is still on the rise [9]. Consequently, an important need exists for alternative therapeutic strategies to prevent or manage hypertension.

Lifestyle interventions that have been shown to prevent hypertension include maintaining a healthy body weight, taking moderate physical activity, eating a low-sodium diet, reducing alcohol consumption, and adherence to a diet rich in fruit and vegetables [10]. Another emerging potential therapeutic lifestyle strategy to prevent or manage hypertension is increasing dietary protein intake [11, 12]. Both increases in dietary protein intake as a macronutrient [12–16], and as smaller doses as bioactive peptides [17–19], have shown the potential to reduce BP. Indeed, evidence exists that plant-based protein sources may be more effective than animal sources; however, these data stem mainly from observational trials and are inconsistent [11, 20]. Randomized controlled trials comparing plant-based protein sources to animal-based protein sources are limited [12, 21–23]. An essential plant-based protein source that may have anti-hypertensive properties is hemp seed.

Hemp seed (*Cannabis sativa* L.) utilization into foods has been limited due to legislation restricting the cultivation of all *Cannabis* species on account of the presence of varieties high in the psychoactive compound tetrahydrocannabinol (THC). However, as of 1998 in Canada, cultivation of hemp seed containing < 0.3% THC has been allowed. Despite heightened consumer demand for hemp food products, due to historical restrictions on the growing of hemp, the nutritional science literature on hemp-seed protein (HSP) remains limited [24]. HSP has a unique amino-acid profile, with high levels of arginine, which makes HSP an ideal protein to consume for BP-reducing effect, or from which to make BP-reducing bioactive peptides [25].

Both observational and intervention trials have identified associations between dietary protein intake and BP [11, 12, 22]. A meta-analysis of 40 randomized controlled trials demonstrated that replacing a median of 40 g/day of dietary carbohydrate with dietary protein is associated with small reductions in both systolic − 1.76 mmHg (95% confidence interval (CI) − 2.33, − 1.20) and diastolic − 1.15 mmHg (95% CI − 1.59, − 0.71) BP (SBP and DBP, respectively) [22]. The meta-analysis did not find a difference between animal- and plant-based-protein sources in terms of BP-lowering effect. However, trials specifically comparing animal proteins to vegetable proteins are limited and have inconsistent results.

The anti-hypertensive effects of protein may not only be related to the amount of protein in the diet but may also be related to the type of protein. Whether a protein is animal or plant based is likely not as important as the type of amino acids contained in the protein source in terms of BP-lowering ability. Animal sources of protein are considered complete proteins, in that they contain an adequate proportion of all nine essential amino acids necessary for human dietary needs. Most vegetable sources individually are not considered complete because they are limited in one or more of the essential amino acids. This is important because the specific amino-acid content of a protein is likely critical to its anti-hypertensive potential. Additionally, different protein sources may affect BP through alternate mechanisms depending on their amino-acid content. Specific amino acids which have been identified as having potential BP-reducing properties include arginine, cysteine, tryptophan and glutamic acid [26].

Casein is the main protein found in cow’s milk [27], with whey protein making up most of the remaining protein. Pal and Ellis [28] investigated the effects of consuming casein or whey protein, compared to glucose, for 12 weeks in 70 men and women using a randomized, parallel-designed trial. Whey- and casein-protein consumption lowered SBP and DBP compared to baseline after 12 weeks and lowered DBP compared to the glucose control. No difference in BP was seen between the two milk-protein fractions. Soybean protein, which has been well-studied for its potential BP-reducing effects [15, 16, 29], is a representative vegetable protein with a high arginine content. This high arginine content may be responsible for some of soybean protein’s anti-hypertensive properties [26]. Unlike soybean, HSP consumption has not been well-characterized in terms of its effects on BP. HSP has even higher concentrations of arginine than soy, casein or whey protein, which may make it an ideal candidate to evaluate for BP control [30]. Consumer demand for hemp food products is growing and health food retailers are looking to add hemp foods to their product offerings. However, hemp foods, unlike casein, whey and soy, have a very limited database of peer-reviewed publications [31–34] outlining the health benefits of consumption due to restrictions in hemp cultivation, which have only recently been lifted in many countries.

Arginine, conditionally an essential amino acid depending on age and health [35], has been shown to reduce BP when supplemented in the diet [35, 36]. The main source of endogenous nitric oxide (NO) is L-arginine, which is oxidized to NO and citrulline by the action of endothelial NO synthase (eNOS) [37]. However, some proteins with incorporated arginine can undergo methylation and, with subsequent proteolysis, can yield methyl-arginine compounds such as asymmetric dimethylarginine (ADMA). ADMA is an endogenous inhibitor of NO synthase and has been shown to reduce the sensitivity of NO synthase to L-arginine. However, ADMA inhibition of NO synthase may be overcome with increased dietary arginine consumption. Additionally, arginine rapidly undergoes catabolism by arginase, a metalloenzyme, which hydrolyzes L-arginine to L-ornithine and urea [38]. Arginase is, therefore, a key regulator of NO availability, and arginase inhibitors are being investigated for their therapeutic potential in the treatment of endothelial function and hypertension [39, 40]. As with ADMA, additional dietary arginine consumption may be able to overcome arginase activity, which limits arginine availability for eNOS and NO production.

Typical sources of dietary arginine are meat, fish and poultry; however, soy protein isolate and HSP also possess higher levels of this amino acid [30, 41], while milk protein is relatively low in arginine [42]. Food-based arginine is well-tolerated with few side effects, making it a better source of arginine than free L-arginine which has been associated with nausea, gastrointestinal discomfort and diarrhea [37]. HSP, due to its high arginine content, is, therefore, a protein source, which should be investigated for its anti-hypertensive effects.

Girgih et al. [25] compared the effects of HSP isolate on the prevention and treatment of hypertension in spontaneously hypertensive rats (SHRs). The replacement of 1% casein on a dry-weight basis in the rat diet with HSP isolate was able to prevent the increase in SBP seen in young growing SHRs over 8 weeks and decreased SBP in adult SHRs with established hypertension. Decreased plasma renin concentrations and angiotensin-converting enzyme (ACE) activity were observed with the HSP feeding. Additionally, Girgih et al. [25] produced an HSP hydrolysate from the enzymatic hydrolysis of HSP isolate. Protein hydrolysates contain bioactive peptides, which are typically produced by enzymatic hydrolysis of food proteins to release short peptide sequences [43]. However, bioactive peptides can also be produced endogenously through the digestion of dietary proteins [19]. Numerous bioactive peptides have been shown to modulate RAAS via inhibition of ACE and renin activity [44]. The HSP-derived bioactive peptides made by Girgih et al. [25] were able to lower SBP in adult SHRs with established hypertension and decreased SBP in young growing SHRs to a greater extent than the HSP isolate when substituted for casein at 1%. The HSP-derived bioactive peptides also reduced renin concentrations and ACE activity more than did the HSP isolate in the SHRs, and even reduced plasma ACE activity in the normotensive control rats.

Since there was no difference in the degree of reduction in SBP in the adult SHRs between HSP isolate and bioactive peptide, but the reduction in ACE activity was much greater with the bioactive peptide [45], it was hypothesized that the intake of HSP isolate was likely lowering BP via a different mechanism than the peptide [25]. Indeed, the marked difference in the arginine content between the HSP isolate and the hemp seed protein hydrolysate (HPH) could be responsible for the differing hypotensive mechanisms. The HSP isolate contained more than six times the arginine in the HSH-derived bioactive peptides [25]. However, none of the rats were fed a combination of the HSP isolate with additional HSP bioactive peptides to investigate whether the BP-reducing effects would be additive or synergistic.

Bioactive peptides are specific, short amino-acid sequences that remain inactive when bonded to other amino acids within the primary structure of a food protein, but after release via hydrolysis, the free forms of these peptides can affect biological processes. Bioactive peptides have been shown to act as anti-hypertensive agents by inhibiting in vitro and in vivo renin, ACE and angiotensin-II receptor activities in addition to enhancing blood NO levels [44]. Therefore, there is an interest in characterizing these natural anti-hypertensive compounds so that they can serve as alternatives to anti-hypertensive drugs. Bioactive peptides have the advantage of specificity, with limited side effects, potency and low toxicity such that they can be consumed at high doses [44, 46, 47]. Different types of bioactive peptides, derived from numerous sources including dairy, meat, fish, poultry, canola, buckwheat, algae and hemp, have shown hypotensive effects in animal models [43, 44].

However, only a limited number of the bioactive peptides that have been characterized in animals and in vivo have subsequently been tested in human trials [19]. Most human trials investigating dietary bioactive peptides have been derived from dairy products, with the results from casein- and whey-derived bioactive-peptide feeding trials being mixed [19, 48]. A meta-analysis of human trials investigating dairy-derived bioactive peptides found that the consumption of 2–5 g of bioactive peptides a day could yield approximately 5-mmHg and 2-mmHg reductions in SBP and DBP, respectively. It has been suggested that the amino-acid composition of the bioactive peptides may be critical to the ability of the peptide to inhibit ACE activity. Bioactive peptides with a positive charge due to arginine or lysine residues are thought to be being beneficial for ACE inhibition [49]. A 3-week crossover human intervention trial conducted by our team fed 3 g per day of pea-protein hydrolysate and led to a 5–6 mmHg reduction in SBP in the second and third weeks compared to placebo [18]. To our knowledge the addition of bioactive peptides on top of increased dietary protein has not been previously investigated in humans.

While there is good evidence that replacement of dietary carbohydrate with protein has anti- hypertensive effects [11–13, 22], numerous gaps in the research still remain. First, comparisons of plant versus animal sources of dietary protein, or high-arginine versus low-arginine sources of dietary protein are limited [11, 22]. Second, the effects of additional dietary protein, where it does not explicitly replace carbohydrate, is not known [22]. Third, human trials investigating HSP consumption are limited [32]. Fourth, human trials involving non-dairy-derived bioactive peptides are limited [19]. Lastly, no trials investigating the potential benefit of increased dietary protein plus bioactive peptides have been completed to our knowledge. To address these knowledge gaps, we propose a clinical trial that would evaluate the anti-hypertensive potential of consuming whole HSP, and HSP+, compared to casein protein using a randomized, double-blinded and crossover design. This design should be able to address the knowledge gaps identified above. Although little work has been done in humans in this specific area, for the following reasons we were not able to perform a pilot trial before the main study; the long-term nature of this study was the main factor that prevented us considering a pilot study as the first phase. The recruitment of 35 participants and obtaining their consent to enter a long-term trial was a challenge so, recruitment of more people as a pilot group would increase the duration of study. So, we had to recruit more people to start the main study or have the pilot group on an extra washout period to be able to start the trial over. These participants might lose their interest in continuing with the study and the drop-out rate would increase. In terms of testing the procedure, we mainly followed similar clinical trials with other treatments to evaluate the acceptability of the procedure in the following criteria; treatment consumption frequency, treatment consumption under supervision, free-living trial with basic recommendations for physical activity and alcohol consumption, method and frequency of the outcome measurements, method of randomization and follow-up with participants. Acceptability of the treatments were assessed using a taste and texture testing with random participants. Sample size was calculated based on the expected change in the main outcome using similar studies considered 24-h SBP as their main outcome.

## Methods/design

### Objectives and hypothesis

The aim of this study is to assess the hypotensive potential of consuming whole HSP, and HSP+, compared to casein protein in patients with hypertension. The hypotheses to be tested in this study are as follows: Hypothesis 1: consumption of 50 g/day of dietary protein, of any type (casein, HSP or HSP+) for 6 weeks will lead to reduced 24-h SBP compared to baseline. Hypothesis 2: consumption of 50 g/day of hemp-based dietary protein (HSP and HSP+) for 6 weeks will reduce 24-h SBP compared to casein. Hypothesis 3: consumption of 45 g/day hemp protein with an added 5 g/day of HSP+ for 6 weeks will reduce 24-h SBP compared to 50 g/day of HSP.

### Study design

**Fig. 1:**
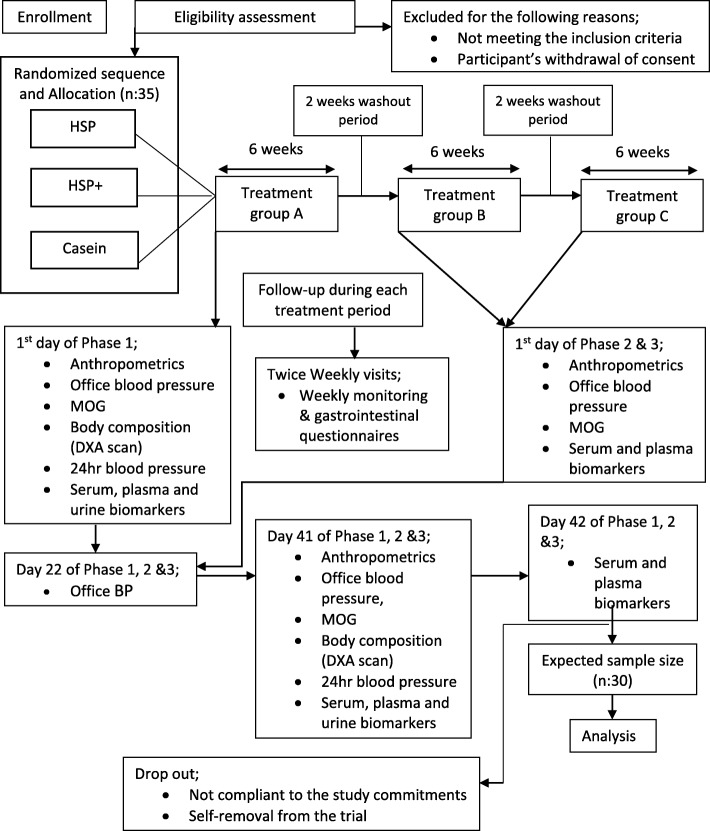
Schematic flow diagram of the trial protocol

**Fig. 2:**
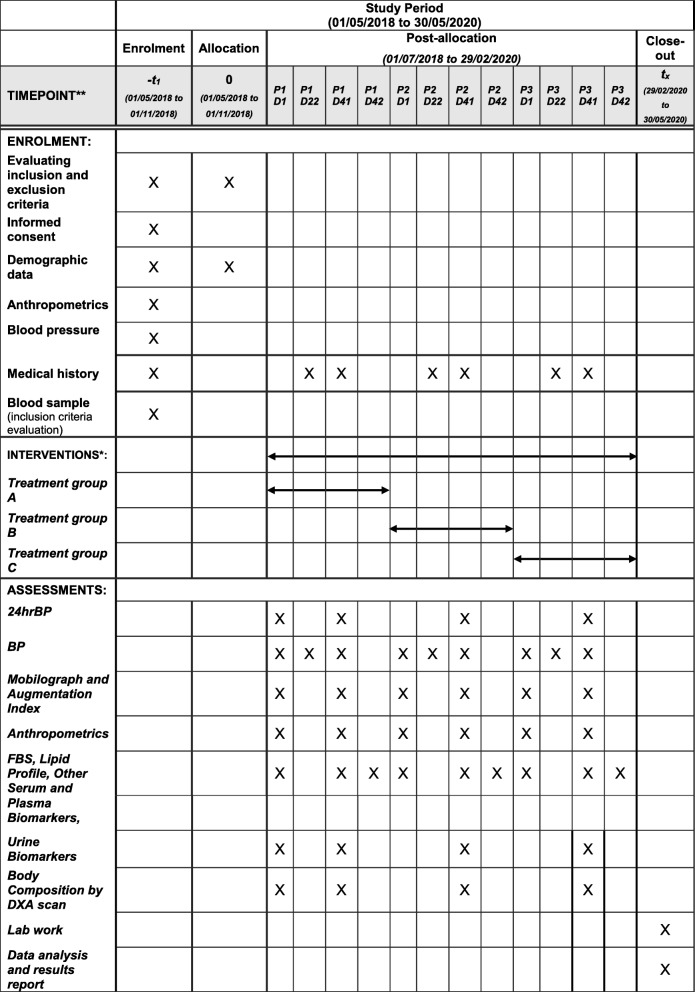
Representation of the trial enrollment, interventions and analysis

An interventional, randomized, double-blind, crossover, phase-II clinical trial is being conducted at the Clinical Nutrition Research Unit at the Richardson Centre for Functional Foods and Nutraceuticals (RCFFN), University of Manitoba. The trial will consist of three treatment periods of 6 weeks each, separated by 2-week washout periods. A summary of the proposed trial design is presented in Fig. 1. The Standard Protocol Items: Recommendations for Interventional Trials (SPIRIT) Checklist is presented in Fig. 2 and Additional file 1.

During each treatment period, participants need to come to the RCFFN twice a week on Tuesdays and Fridays to consume one treatment under supervision and pick up treatments for the next 2 or 3 days. Each treatment consists of a frozen fruit smoothie (mango or pineapple), fruit juice (diet mango juice), sorbet and 25 g of protein from treatment-protein powder and the treatments will be consumed twice a day. HSP and the HSP+ powder has a green color and a stronger taste than casein-protein powder; however, our team has identified that by using a combination of frozen fruit and sorbet in the fruit smoothies this color and taste can be well-masked.

Participants will be instructed to consume two or less alcoholic beverages and no more than three 8-oz. cups of caffeine-containing beverages per day during the trial period. Participants will be instructed to perform their normal daily activities during the measurement day. Prolonged intense physical activity will be discouraged on measurement days and the days preceding measurement days. Participants will be strongly recommended to maintain consistency in their physical activities and dietary pattern during the trial period. A 3-day food record will be collected during the first day of the first phase and during the final week of each treatment period to be analyzed for calorie intake during the study.

During the study period, body weight will be monitored as a change could occur due to the inclusion of extra protein in a free-living condition. All outcome variables will be adjusted for changes in body weight should significant changes occur between treatments. Gastrointestinal tolerability and weekly monitoring questionnaires will be completed by participants weekly during each intervention period.

### Sample size

A total sample size of 30 participants was calculated as required to detect a change in 4 mmHg in SBP with the proposed crossover design, using an estimated within-participant standard deviation of 5.45 mmHg and a power of 0.80 and *α* = 0.05 [13]. A recruitment goal of 35 participants has been set to account for the potential participant drop out.

### Study setting

This study will be conducted at one site the RCFFN, University of Manitoba, Winnipeg, Manitoba, Canada R3T 2 N2 (Winnipeg is the capital and largest city of the province of Manitoba, with a population of approximately 775,000 people, of primary European decent, but with a significant Indigenous and Filipino population). The site investigator is Dr. Rotimi Aluko at the RCFFN, University of Manitoba.

### Study population

Thirty-five participants will be enrolled into the study using advertisements in local media including social media, newspapers and the official website of the Richardson Centre and on University Campuses, as well as our pre-existing database of participants. Potential participants will be initially screened over the telephone using a telephone questionnaire where some brief questions regarding personal health information will be asked. If participants are determined as potentially eligible after telephone screening, they will be scheduled to come to the RCFFN in the morning to have their BP measured and undergo a blood screening where 20 ml fasting blood samples will be taken via venipuncture by a nurse in order to test for blood parameters related to the exclusion criteria. If the potential participants meet all the inclusion criteria before they go for the blood draw and not the exclusion criteria, the informed consent form will be provided by the study coordinator and the participant will sign the form and will be recruited to the study.

### Inclusion criteria

Hypertensive participants aged 18–75 years will be recruited, with a Body Mass Index (BMI) between 18.5 and 40 kg/m^2^. Participants must have a SBP between 130 and 160 mmHg and a DBP ≤ 110 mmHg. Additionally, participants must be willing to fast for 10–12 h and abstain from alcohol for 2 days prior to blood sampling and BP measurement. They must also abstain from coffee consumption for at least 14 h and physical exercise for at least 4 h before BP measurement. All women of child-bearing age will take a pregnancy test prior to commencement of the trial. The pregnancy test should be negative for women with child-bearing potential. All participants must be able and willing to give informed consent to participate in the trial prior to their inclusion.

### Exclusion criteria

Participants will be excluded if they are undergoing change in, or have changed, the type or dosage of anti-hypertensive drug treatment, regular high-dose non-steroidal anti-inflammatory drug (NSAID) treatment, cyclosporine or tacrolimus in their treatment for less than 3 months. Participants will be excluded if they have any dietary restrictions, which would prevent them from consuming the trial treatments. Participants must not have self-reported weight gain or loss greater than 5 kg in the past 3 months. Participants must be free of active cardiovascular disease including stroke, congestive heart failure, myocardial infarction, unstable angina pectoris, coronary artery bypass graft, percutaneous transluminal coronary angioplasty, temporal ischemic attacks, anemia, abnormal electrolytes, proteinuria, abnormal liver, kidney, thyroid function, bleeding/blood or psychiatric disorder. There will be some exceptions regarding some medications/supplements consumption by the participants. Those participants taking BP medications who have been stable on the medication for at least 3 months will be included in the study. Those taking medications (i.e., statins) or supplements (i.e., fish oils) for lipid lowering reasons and have been stable on such medications and supplements for at least 3 months will be included in the study. Participants with stabilized thyroid levels with treatment will be allowed to participate in the study.

Participants will be asked about current or historical diseases/conditions through verbal question and answer during screening. Participants will be excluded if they have clinically significant biochemistry defined as: sodium < 134 mmol/l, > 148 mmol/l; fasting glucose > 6.1 mmol/ l or any other clinically significant abnormality in hematology and/or biochemistry at the investigator’s discretion.

Participants will be excluded if they have secondary hypertension, have type 1 or type 2 diabetes, a history of cancer or malignancy in the last 5 years, or any metabolic disease, gastrointestinal disorder or other clinically significant disease/disorder, which could interfere with the results of the study or the safety of the participant or those with allergies or sensitivities to any of the ingredients in the study product.

Participants will be excluded if they smoke tobacco, use snuff, are nicotine users, recreational drug users, or if they consume more than 14 alcoholic beverages a week. Women with child-bearing potential not using an acceptable method of birth control (i.e., implants, injectables, combined oral contraceptives, some intrauterine contraceptive devices (IUDs), sexual abstinence or a vasectomized partner) or who are pregnant or plan to become pregnant during the trial period or women who are breastfeeding will be excluded.

### Conditions for withdrawal from the trial

The following criteria are to be considered sufficient reason for discontinuing participation in the trial: non-compliance by the participant; developing condition(s) or consumption of supplements or medications as specified under the exclusion criteria or changing the type or dosage of the specified medications; presenting an adverse event or any other medical situation where, in the opinion of the qualified investigator, continuation of the trial treatment would compromise the participant’s health; abusive behavior of a participant towards trial staff; participant’s withdrawal of consent and self-removal from the trial for any reason. We will follow up with study participants who have been withdrawn from the trial due to an adverse event, until symptoms have resolved.

### Randomization and concealment procedures

Recruited eligible participants will be randomly assigned to one of the treatment sequences using a 3 × 3 Latin-square design. The letters A, B and C represent the three treatment groups and the sequences are as follows: ABC, ACB, BAC, BCA, CAB and CBA. The randomization procedure will be completed using a random-number generator.

### Blinding

Due to the fact that the casein powder, HSP and HSP+ have different visual appearances, treatment smoothies will be prepared by a kitchen staff member to reduce the differences in treatment appearance and given to the clinical coordinators in a blinded fashion labeled A, B or C. Assignment of treatments to labels will be performed by a researcher independent of the investigators’ groups. This researcher will keep a copy of the treatment coding, give a copy of the treatment coding to the assigned kitchen staff, and give a sealed copy of the coding to the principal investigator allowing participants, coordinators and individuals performing the analyses and collecting outcome data to be blinded to the treatments. Participants will be instructed not to communicate about the taste and texture of the smoothies with the research staff or other participants in an attempt to ensure double-blinding. The treatment coding will be broken after the conclusion of the statistical data analyses on the primary outcome. However, the code could be broken if there is an adverse event reported which requires identification of the treatment.

### Sample collection

Twelve-hour morning fasting (i.e., nothing to eat or drink except water 12 h prior) blood samples will be collected on the first day of each phase (baseline), and on days 41 and 42 of each treatment periods of the trial (in total nine times during the trial). This fasting blood samples (approximately 4 teaspoons or 20 ml) will be taken from the top of the forearm via venipuncture by a phlebotomist on each blood-draw day for assessment of blood biochemical markers. Each blood test will take approximately 5 min. No alcoholic beverages are to be consumed within 48 h prior to blood draws during the study periods. No food or caffeinated beverage consumption is allowed within 12 h prior to blood draws during the study periods. Blood samples will be centrifuged at 3000 rpm for 20 min at 4 °C, aliquoted to yield serum, plasma and RBCs, and then stored at − 80 °C until analyses.

Participants will be asked to collect 24-h urine samples at the first day of first phase and last day of each treatment period. Urine containers will be kept in the fridge during the day of collection, and then will be aliquoted and stored at − 80 °C until analyses. The volume of the 24-h urine samples and start and end time of collection will be recorded.

### Compliance

Compliance will be monitored, regarding participants having consumed the morning treatment smoothies on Tuesdays and Fridays, through visual affirmation by clinical coordinators. Participants will be required to return smoothie containers from other days’ smoothie treatments on the next visit. Also, participants need to fill out a treatment consumption checklist which include the date and timing of each treatment consumption and return the checklist at the end of each treatment period. Missed smoothie consumption or return of smoothie containers will be recorded for each participant. Non-compliance will be defined as missing supervision for, or failing to return the empty smoothie containers from, 80% of the total smoothies per treatment period. Missing two consecutive measurement or blood-sampling days will also be classified as non-compliance. Non-compliant participants will be asked to leave the trial. An intent-to-treat analysis will be performed to use the data from non-compliant participants.

### Study groups

Eligible participants will be assigned randomly to one of the following sequences: ABC, ACB, BAC, BCA, CAB or CBA. So, all the participants will be provided with all three treatments in a crossover design.

### Study period

This study includes three phases of 6 weeks each, separated by two washout periods of 2 weeks. Participants will be involved in the study for a period of 22 weeks.

### Outcomes and measurement tools

The primary outcome of this trial will be 24-h SBP, measured at the first day of the first phase and day 41 of each treatment phase using ambulatory BP monitors (ABPM, OnTrak, Spacelabs Healthcare Inc., Snoqualmie, WA, USA) [50, 51]. Participants will be fitted with an ABPM to wear for a full day. Continuous and SBP and DBP measured over 24 h will be recorded.

Office-measured BP, pulse-wave velocity and augmentation index will be determined using an automated oscillometric measurement device (Mobil-O-Graph, IEM, Stolberg, Germany) in an office setting at days 1 and 41 of each treatment phase [52, 53]. Office-measured BP will also be measured at day 22 to track the changes at the midpoint of each treatment phase. This measurement will take place in a quiet room while the participant is in a seated position with their arm resting on an arm rest at heart level. Participants will be advised to rest quietly throughout the measurements. Oscillometric measurement will be performed four times at 2-min intervals. The first measurement will be discarded, and the last three measurements will be averaged to determine office-measured BP. The LCD display of the oscillometric measurement device will be covered so that the participant is blinded to the BP values during the measurements. BP results will not be shared with participants until they have completed the trial.

### Anthropometric measurements

At days 1 and 41 of each treatment period, body weight and waist and hip circumferences will be measured following standardized procedures. Body composition will also be assessed by dual x-ray absorptiometry (DXA) scan at the first day of the first phase and day 41 of each treatment period (in total four times during the study) to look at potential changes in body fat and lean mass composition. For this procedure, the participant will need to lie in a horizontal position for about 5–15 min while the scan arm passes from the head to the feet.

### Blood biochemical measurements

Serum samples will be analyzed for the following: fasting lipid profiles including total cholesterol (TC), high-density lipoprotein (HDL-C), low-density lipoprotein (LDL-C) and triglyceride (TG) levels, as well as, glucose, serum creatinine, blood urea nitrogen (BUN), aspartate aminotransferase (AST), alanine aminotransferase (ALT), gamma-glutamyl transferase (GGT), serum total protein and serum albumin will be measured using the automated enzymatic methods on a cobas c 311 analyzer (Roche Diagnostics, Indianapolis, IN, USA). Fasting plasma insulin concentrations will be determined using radioimmunoassay (RIA) (EMDMillipore, Etobicoke, ON, Canada) [54]. Insulin homeostasis modelling assessment (HOMA) will be utilized as an estimate for % β-cell function and insulin resistance (IR) [55].

ACE activity in the plasma will be measured by a spectrophotometric method using FAPGG as substrate [25]. Serum angiotensin II, serum aldosterone, renin, epinephrine and norepinephrine will be measured by commercial enzyme-linked immunosorbent assays (ELISA). NO concentrations will be determined in plasma using a colorimetric kit (Nitric oxide assay, Thermoscientific, Waltham, MA, USA) [56].

### Urine biochemical measurements

Twenty-four-hour urinary sodium, potassium, creatinine, magnesium, total protein and albumin will be measured on a cobas c 311 analyzer, (Roche Diagnostics, Indianapolis, IN, USA). NO concentrations will be determined in urine using a colorimetric kit (Nitric oxide assay, Thermoscientific, Waltham, MA, USA). Urinary epinephrine and norepinephrine concentrations will be measured by ELISA.

### Statistical analysis

Statistical analysis will be performed in an intent-to-treat fashion. In addition, a per-protocol analysis will also be performed to check for potential effect attenuation due to unequal compliance or protocol deviations across treatments. Statistical analysis will be performed using SAS 9.4. The endpoint of treatments and baselines will be compared using a mixed-model analysis of variance. Sequence and sex will be included as fixed factors, and participant will be included as a random factor, with participant repeated by period.

Dunnett’s test will be used to adjust for multiple comparisons with the endpoint of the baselines set as the control. Effects of treatment by time on measures repeated during treatments will be assessed using a mixed-model analysis of variance with sequence, sex, time as fixed factors and participants included as random factors, with participants repeated by period, with Tukey-Kramer adjustments for multiple comparisons. Model residuals will be visually inspected to assess independence, normal distribution, and constant variance. Datasets with residuals determined to violate these assumptions will be transformed prior to re-analysis. Results will be expressed as estimated least square means ± standard error of the mean (SEM) for all values. Statistical significance will be set at *p* < 0.05 for all analyses.

### Risks and benefits

No serious side effects or adverse events have been previously reported due to hemp or casein protein intake.

Phlebotomy carries a potential health risk. DXA scans expose participants to x-rays. Participants will be encouraged to report any adverse events as they happen, and participants will be prompted and asked on a weekly basis about adverse events. Adverse events will be recorded through the “Adverse Event/Unanticipated Problems Form” provided by the Research Ethics Board (REB). All serious adverse events will be reported to the University of Manitoba REB which has approved the trial protocol. Study coordinators will follow up with participants via email or telephone after adverse events until symptoms have resolved. If the serious unexpected adverse reaction (SAEs) is fatal or life-threatening, the Therapeutics Products Directorate of Health Canada will be notified no later than 7 days after the sponsor becomes aware of the information. If the serious unexpected adverse reaction is neither fatal nor life-threatening, the Natural and Non-prescription Health Products Directorate (NNHPD) will be notified immediately if possible, and no later 15 days after the sponsor becomes aware of the information. Within 8 days after having informed the NNHPD of a serious unexpected adverse reaction to the NHP, the sponsor will submit a report as complete as possible that includes an assessment of the importance and implication of any findings. The final report will include relevant previous experience with the same or similar health products. The trial will be conducted following the principles of Good Clinical Practice.

### Concomitant therapy

All prescription and over-the-counter medications that are not deemed as falling into the exclusion criteria list will be recorded at the screening visit by a general physician and will be monitored on a weekly basis during the treatment periods. Should a medication be prescribed or started during the trial period, it will require qualified investigator approval.

### Anticipated results and conclusion

It is anticipated that the additional 50 g/day of dietary protein, whether from casein or HSP, or HSP+, will result in a reduction in BP compared to the baseline of the trial. Such results would support a simplified message that increasing dietary protein, by consuming 50 g per day of protein through the use of protein powders, can help lower BP. Such a recommendation is easier to explain and implement than recommendations where a specific macro-nutrient like carbohydrate or fat must be replaced by protein to have anti-hypertensive effects.

It is also anticipated that the hemp protein treatments will lead to a greater reduction in BP compared to the casein, due to their increased arginine concentration. These results will be accompanied by increased blood NO concentrations on the hemp protein treatments. This finding would suggest that HSP, or protein sources with higher arginine, are appropriate choices of protein to select for individuals who may be looking to control their BP rather than lower arginine dietary protein sources.

The HSP+ can be predicted to lead to the greatest reduction in BP. The HSP+ treatment is predicted to demonstrate the greatest reduction in ACE activity due to the ACE-inhibitory effects of the bioactive peptides. If an additional benefit is seen with the addition of HSP bioactive peptide to the HSP, this finding would suggest that dietary protein and bioactive peptides lower BP through separate or synergistic mechanisms, and that a combination of increased dietary protein and bioactive peptides could be recommended for greater BP reduction over and above a simple increase in dietary protein intake.

This proposed trial should directly address gaps in the scientific literature surrounding the anti-hypertensive properties of dietary protein. An increased understanding and demonstration of the anti-hypertensive properties of dietary protein and food-protein-derived bioactive peptides is required before greater recommendations of increased dietary protein can be made to individuals who are looking to control their BP without resorting to pharmaceutical agents.

### Participant privacy and confidentiality of data

The following information will be collected during the course of this project: name, address, date of birth, primary telephone number, and sex. This information is recorded (hard copy with scanned electronic back-up copy), which also contains the individual’s written consent to participate in the project. Study records that contain identities will be treated as confidential in accordance with the Personal Health Information Act of Manitoba. These forms will be stored in a locked file cabinet in a locked office in the RCFFN and will never leave the site. The electronic back-ups will be on a single password-protected computer. The contact information will be entered into a separate contact Excel spreadsheet that contains no other personal/health information and will only be used by the study coordinator for the purpose of informing/updating study participants while the study is being carried out. This file is maintained on a single computer and will be password encrypted. The study data will be stored for 25 years after the completion of the study. All paper materials and samples related to the study will be stored in the locked filing cabinet in a locked office or locked freezers at the RCFFN under the supervision of Dr. Rotimi Aluko. Only the study coordinators and the principal investigator will have access to the samples. All study documents will bear an assigned participant code. If the results of the trial are published, the identity will remain confidential. All study data will be labeled with only the assigned participant code. This unique code will only be known to the participant and the research study staff. Documents of personal information linking the participants to their codes will be kept separately from any other records, also in a secure locked area. Due to the coding system, there will be no link between personal data and the sample data collected during analysis. All print materials will be destroyed using the contracted document destruction service at the University of Manitoba. All electronic files will be deleted in an encrypted format.

Health Canada may review and research the records for auditing purposes. The University of Manitoba Biomedical REB may review research-related records for quality assurance purposes. Organizations that may also inspect/copy the research records for quality assurance and data analysis include groups such as: Health Canada, their representatives, and other researcher groups who are performing meta-analysis or knowledge synthesis work who request access to the raw data. This review or use of raw data will not include personal information such as name, address, telephone number and/or any other identifying information. Any data required to support the protocol can be supplied on request.

## Discussion

Unlike earlier trials which have compared carbohydrate to protein in terms of BP, the currently proposed trial will compare different types of protein added into the participants’ diet. In this instance, the participant may or may not replace existing calories in the diet with the additional protein. Therefore, the potential exists that increased protein consumption will lead to a change in overall energy intake. An increase in energy intake could occur if the additional calories of the protein are not being compensated for by participants, or a decrease in energy intake could occur due to the satiating properties of protein compared to other macronutrients. These potential shifts in overall energy intake could result in changes in body weight, which could lead to BP changes. Therefore, outcome variables, such as BP, will be adjusted for any significant changes in body weight, which may occur during the treatment periods. Additionally, the mix of fat and carbohydrate being potentially replaced by protein in terms of energy intake may vary between participants. While this trial will not be able to determine exactly whether fat or carbohydrate energy is being replaced with protein, it will reflect the real-world impact of instructing patients and the general public to increase protein consumption by drinking protein smoothies, without specific counseling on replacing other foods.

As in all clinical trials with food-based treatments, potential differences in taste, texture and smell can make the blinding of participants to the treatments difficult. Our research team has worked with HSP and has developed a smoothie recipe which is good at masking the type of protein powder from which it is made. However, if this blinding is not sufficient it may be possible for participants to identify the casein treatment versus the HSP and HSP+ treatments. The likelihood of participants being able to identify the HSP compared to the HSP+ treatment is low. Fortunately given the crossover design and the stringent monitoring of compliance in the proposed trial, it is unlikely that any un-blinding of the participants to the treatments, as described above, would be able to impact the results. All of the outcomes in this trial will be objectively measured; therefore, the impact of knowing if you are on a hemp-based treatment is unlikely to change your outcome measurements.

Knowing you are on a particular treatment, for which your compliance is monitored and controlled, is unlikely to influence your BP measurement, especially given the fact that BP measures will not be shared with participants until they have completed the entire trial.

### Trial status

Recruiting. The date of starting recruitment for this study was 1 May 2018 and the approximate date when recruitment will be completed is 29 February 2020. The current protocol version number is 6.

### Trial Monitoring

#### Trial Steering Committee (TSC) and Data Management Team

Dr. Rotimi E. Aluko (principal investigator and project lead) and Maryam Samsamikor (clinical coordinator, responsible for recruitment and consent process), will administer the study. Any changes in the protocol, testing or recruitment criteria will be communicated to the Bannatyne REB of the University of Manitoba through amendments. Also, all the amendments will be approved by the NNHPD branch of Health Canada. The REB will receive an annual progress report of the study and its team will supervise the process of conducting the research according to the protocol and quality of confidentiality where needed.

### Protocol modification

Any changes in the protocol will be first approved by the principal investigator of the study. Then, a detailed amendment will be sent to the University of Manitoba REB for conditional approval. The final approval from the REB will be obtained after submitting the approval from the NNHPD of Health Canada to the REB for the related changes. Before all approvals are granted, no changes in the procedure will be made. The University of Manitoba REB will audit the study and any deviations from the protocol will be documented as a breach report. The clinical trial registry will be updated according to any changes made to the study protocol.

### Availability of data

Biological specimens, such as blood and urine samples, will be kept in the freezer until further analysis. There will be no analysis other than what the participants will be aware of and will give the consent for. The results of this study will not be shared as a dataset but non-identifying results will be posted on the World Wide Web for other researchers to use. Also, the results at the participant’s level will be shared with them when available.

## Supplementary information

**Additional file 1.** Standard Protocol Items: Recommendations for Interventional Trials (SPIRIT) 2013 Checklist: recommended items to address in a clinical trial protocol and related documents.

## Data Availability

The datasets used and/or analyzed during the current study will be available from the corresponding author on reasonable request following the completion of the trial.

## Abbreviations

ACE: Angiotensin-converting enzyme
ADMA: Asymmetric dimethylarginine
ALT: Alanine aminotransferase
AST: Aspartate aminotransferase
BUN: Blood urea nitrogen
CI: Confidence interval
DBP: Diastolic blood pressure
DXA: Dual x-ray absorptiometry
ELISA: Enzyme-linked immunosorbent assay
FBS: Fasting blood sugar
GGT: Gamma-glutamyl transferase
HDL: High-density lipoprotein
HSP: Hemp seed protein
HSP+: HSP plus HSP hydrolysate
IR: Insulin resistance
IUDs: Intrauterine contraceptive devices
LDL: Low-density lipoprotein
NNHPD: Natural and Non-prescription Health Products Directorate
NO: Nitric oxide
RAAS: Renin-angiotensin-aldosterone system
RCFFN: Richardson Centre for Functional Foods and Nutraceuticals
SBP: Systolic blood pressure
SEM: Standard error of the mean
SHRs: Spontaneously hypertensive rats
SPIRIT: Standard Protocol Items: Recommendations for Interventional Trials
TC: Total cholesterol
TG: Triglycerides
THC: Tetrahydrocannabinol
